# Unlocking the promise of UK health data: considering the case for a charitable GP data trust

**DOI:** 10.1093/medlaw/fwae043

**Published:** 2024-12-10

**Authors:** Caroline A B Redhead, Catherine Bowden, John Ainsworth, Nigel Burns, James Cunningham, Søren Holm, Sarah Devaney

**Affiliations:** Centre for Social Ethics and Policy, Law Department, The University of Manchester, Oxford Rd, Manchester, M13 9PL, United Kingdom; Centre for Social Ethics and Policy, Law Department, The University of Manchester, Oxford Rd, Manchester, M13 9PL, United Kingdom; Division of Informatics, Imaging & Data Sciences, School of Health Sciences, The University of Manchester , Oxford Road, Manchester, M13 9PL, United Kingdom; Centre for Social Ethics and Policy, Law Department, The University of Manchester, Oxford Rd, Manchester, M13 9PL, United Kingdom; Division of Informatics, Imaging & Data Sciences, School of Health Sciences, The University of Manchester , Oxford Road, Manchester, M13 9PL, United Kingdom; Centre for Social Ethics and Policy, Law Department, The University of Manchester, Oxford Rd, Manchester, M13 9PL, United Kingdom; Centre for Social Ethics and Policy, Law Department, The University of Manchester, Oxford Rd, Manchester, M13 9PL, United Kingdom

**Keywords:** Charitable Incorporated Organisation, data stewardship models, data trust, general practice data trust, health research, patient choice

## Abstract

The UK National Health Service general practice (GP) patient data constitute a rich research resource, but collecting, managing, and sharing patient data present challenges. In May 2021, to address these challenges, substantial changes to the system for processing pseudonymized GP patient data in England were announced. As part of an opt-out process, patient consent to sharing GP data was deemed to have been given. However, when over a million people quickly acted to opt out of the new system, the process was paused, and an engagement exercise commenced, whose aim was to inform a re-designed programme addressing patient concerns. In this article, we present and discuss the findings of the General Practice Data Trust pilot study, which has investigated people’s reasons for opting out of sharing their data, and, looking for practical solutions to their concerns, has discussed with participants the concept of a ‘data trust’ to manage the sharing of patient data. Making a conceptual argument for the use of the (relatively new) charitable incorporated organization as a governance model for a GP data trust, we demonstrate how this could address patients’ concerns and represent a more attractive means of stewarding GP data for research and service planning purposes.

## I. INTRODUCTION

*73 years of NHS patient records contain all the noise from millions of lives. Perfect, subtle signals can be coaxed from this data, and those signals go far beyond mere academic curiosity: they represent deeply buried treasure, that can help prevent suffering and death, around the planet, on a biblical scale*.^1^

The UK National Health Service (NHS) data are considered a research resource of global importance.^2^ Its use for therapeutic and service research and development represents an opportunity both to improve NHS care and to drive innovation in the life sciences sector. NHS data are rich because the NHS population is larger and more ethnically diverse than other countries with similarly detailed health records.^3^ As a research resource, it can be used to discover which treatments work best in which patients. It can indicate which treatments have side effects, and what and how harmful they are. It can be used to help monitor and improve the quality, safety, and efficiency not only of NHS services in the UK but also of health services elsewhere. But, in order to fulfil that potential, NHS data must be checked and shaped, housed and managed securely, analysed, communicated, and acted upon. That work requires people, systems, and platforms that work efficiently and, crucially, in a way that attends appropriately to patients’ concerns regarding the handling of their health data (relating, eg, to their dignity and privacy).

In May 2021, intending to make progress towards realization of these aims, NHS Digital, the (then) national custodian for health and care data in England,^4^ announced that substantial changes were to be made to the collection and use of patient data from general practices (GPs) in England. Part of primary care services, which act as the ‘front door’ of the NHS, GP doctors often represent a patient’s first point of contact with the healthcare system. On 12 May, by way of a Data Provision Notice,^5^ NHS Digital informed GPs that the ageing General Practice Extraction Service was ‘unsustainable going forward’ and would be replaced by a new information system to support the collection and analysis of GP data.^6^ It was proposed that the General Practice Data for Planning and Research (GPDPR) programme would, with patient consent (deemed as part of an opt-out system), underpin the collection and use of pseudonymized GP data to support vital health and care planning and research.^7^ It quickly became clear, however, that a significant number of patients did not consent. More than a million people acted to opt out of the GPDPR programme in 1 month.^8^ In an unambiguous confirmation of the potential value locked up in GP health data (and evidencing an exception to the general perception that data ‘subjects’^9^ are rarely in a position to bargain), NHS Digital reacted by delaying the start of the GPDPR programme. Instead, it signalled an intention to ‘listen, understand, engage and act on what we learn to get this right’.^10^ NHS Digital committed, as part of this process of engagement, to re-design the programme ‘from the ground up’, and to work with health and care professionals, including the Royal College of GPs, research organizations and patient charities, data experts, and the National Data Guardian, patients, and the public to establish how best to address the concerns raised.^11^

Research shows that, in general, people support the idea of sharing their data to improve healthcare.^12^ However, the scale of the opt-out from NHS Digital’s GPDPR programme indicates that significant concerns must be addressed to persuade patients to contribute their data to such a project. In this article, we present and discuss the findings of the General Practice Data Trust pilot study (GPDT study), whose researchers engaged with patients and the public to explore and understand their concerns.^13^ The research investigated people’s reasons for opting out of sharing their GP data and, looking for practical solutions to the concerns identified, advanced and discussed with participants the concept of a ‘data trust’, defined as a legal structure that provides independent stewardship of data^14^ to manage the sharing of their GP data.

The concept of the data trust represents a distinct departure from the use of established data-control mechanisms, including consent, legislation, and the use of regulatory oversight to set and enforce agreed minimum requirements for the legitimate and transparent processing of personal (and other) data.^15^ As the name suggests, a data trust is an adaptation of an equally well-established legal framework, by reference to which one party authorizes another to make decisions about some type of property for the benefit of an identified group of beneficiaries. A data trust represents a similar approach to looking after and making decisions about *data*: it is an institution specifically designed and set up to govern data (including, but not limited to, personal data) on behalf of those who have agreed to share them.^16^ The trustee of a data trust, an independent person, group, or entity with responsibility for stewarding the data, takes on a fiduciary duty, considered the highest level of obligation that one party can owe to another,^17^ to the beneficiaries of the data trust. In this context, that duty involves stewarding data with impartiality, prudence, transparency, and undivided loyalty.^18^ We can, thus, characterize the use of a data trust as something distinctively different from other attempts to address patients’ concerns and researchers’ frustrations in the context of the use of NHS data for medical research and planning purposes.

The findings of the GPDT study are discussed in more detail elsewhere.^19^ In this article, we make a conceptual argument for the use of a charitable legal structure as an organizational model for a GP data trust. We demonstrate how a charitable model could address the concerns of those who opted out of the GPDPR programme and represent a more attractive means of supporting the use of NHS GP data for research and service planning purposes.^20^

The article proceeds as follows. In Section II, we consider in more detail the value of GP data in the UK. We examine the current challenges with, and barriers to, the sharing of that data for research and planning purposes and briefly review why previous attempts to change the status quo (such as care.data^21^) failed in the past.^22^ One of the aims of the GPDT study, which is introduced in more detail in Section III, was to understand what needs to be done differently this time to release the value of GP data for research. In Section IV, we discuss a possible organizational model for a GP data trust. Considering the nature of a GP data trust’s activities, and the support of our participants for a model that is underpinned by an altruistic motivation to return benefit to the NHS, we consider a charitable model, setting out an argument in favour of a charitable incorporated organization (CIO). We conclude, in Section V, by standing back a little from the detail of the previous sections and briefly taking stock of some of the broader implications of our suggestions, noting the need for further empirical, technical, and practical inquiry, but concluding (subject to the availability of an effective and appropriate technical environment) that a CIO could provide a mechanism to meet patients’ requirements for trust and transparency, and also offer patients some control over sharing their health data for altruistic ends.

## II. THE VALUE OF GP DATA AND ACCESS CHALLENGES IN THE UK

In oral evidence to the Science and Technology Committee of the House of Commons, Professor Ben Goldacre^23^ described GP data as the single most valuable data asset that the NHS has, ‘the jewel in the crown of NHS data’.^24^ In describing it as such, he noted two particular characteristics of GP data that, together, combine to set it apart from other health datasets. The first of these is its granularity. As the first point of contact for most NHS patients, GPs collect detailed information about almost every health service contact, every prescription, blood test, referral, and diagnosis that is recorded in primary care, as well as, in many cases, some information about any additional secondary care provided. GP data are also complete in terms of its population coverage across the UK and represent a rich historical resource. Professor Goldacre suggested that GP data are a unique resource, offering ‘huge opportunities’ for traditional academic research, for the design of improvements to health services, for monitoring the quality, safety, and effectiveness of healthcare, and for driving innovations in the life sciences.^25^

In its recent report on improving access to NHS health data, the Association of the British Pharmaceutical Industry (ABPI) describes, with reference to the process of medicine development, how value is created for health systems and their patients.^26^ Routine health data are the starting point: at its most basic, such routine data offer both the NHS and the biopharmaceutical industry an understanding of the safety and effectiveness of existing medicines and treatment pathways. However, routine health data can also increase understanding of the underlying causes of disease, underpin the development of interventions for detecting disease earlier and assist in understanding particular risk factors, thus enabling patient stratification, and, in turn, more targeted treatments. In addition, analysis of current health data in comparison to *historical* GP data makes it possible to identify unmet needs, which can then become the focus of new research investments—and so the cycle continues. Despite Professor Goldacre’s assessment of the value of NHS data to the cycle of medicines development (with which the ABPI agrees^27^), evidence suggests that an increasing proportion of global research and development investment funding is being placed elsewhere.^28^

Realizing the opportunities represented by the rich GP dataset, however, requires systemic change. Commentators from both the pharmaceutical industry and the academy note that challenges in *accessing* NHS data negatively impact research investment in the UK, suggesting that better access would lead to increased funding and, as a result, incremental and iterative improvements to NHS datasets.^29^ In turn, they argue that the availability of innovative therapies in the UK would increase. All of these things depend on more straightforward and streamlined structures and processes to underpin access. However, as the failure of the GPDPR programme suggested, some patients’ unwillingness to share their data also represents a significant barrier to changing the status quo. Our findings indicate that, while people are willing in principle to share their data, they lack both trust in the system as it currently stands and clarity about how the *value* from health data research is shared with the NHS and, ultimately, its patients.^30^ As a result, their preference is to exercise control by declining to share it.

The research carried out by the GPDT study incorporated participants’ perspectives on what would be important to underpin public trust and engagement, as well as research into the technical mechanisms that could support secure platforms and data sharing mechanisms. The study’s conclusions offer useful suggestions for change.

## III. THE GP DATA TRUST PILOT STUDY

### A. Background

The central aim of the GPDT study was to understand patients’ concerns about GP data sharing and to explore ways to give them greater autonomy in decisions regarding the use of their data for health research and planning. As discussed above, the launch of the GPDPR programme in May 2021 prompted more than a million people to vote with their feet and withdraw their (assumed) consent to the proposed sharing of data. Their collective power, in terms of the value of their aggregated GP data, was sufficient to stop the progression of the government’s policy agenda in its tracks.

The study aimed to explore *why* so many people decided to opt out of sharing their GP data. In particular, it sought to understand the nature of the concerns that underpinned people’s decision to opt out and whether (and, if so, what) features of a GP data trust might provide sufficient reassurance for them to change their minds. The empirical evidence collected would inform and underpin suggestions for the structure and governance of a GP data trust, with the intention being that the data trust ‘solution’ would map exactly onto the GPDPR ‘problem’. The GPDT study did not aim to establish a working data trust but rather to undertake the work required to identify and propose solutions to the key challenges identified. These challenges, and the methodological approaches used to explore them empirically, are briefly described below.

The key challenges were:

- recruiting participants to the GPDT study;
- establishing how relationships between the GP data trust and other stakeholders could work;
- identifying appropriate technical systems to support access to the data; and
- identifying an appropriate operating model.

#### 1. Recruiting participants to the GPDT study

No central database was created of the million plus citizens who opted out of the GPDPR programme. The early work of the GPDT study, therefore, included the deployment of a campaign to reach out to those who had opted out and to recruit them to the project so that we could understand their reasons for opting out, their views on health data sharing in general, and, in particular, their thoughts about the use of a data trust to underpin the sharing of GP data.

#### 2. Establishing how relationships between the GP data trust and other stakeholders might work

A secure interface between GP data trusts and the external organizations with oversight of patients’ medical records would be a fundamental feature of a practically workable system. Key external organizations for a GP data trust would include GP partnerships (as data controllers), other data controllers, and data processors in relation to patient data, including NHS England (with which NHS Digital has now merged).^31^ Significant levels of trust would need to be established for data controllers to engage with the GP data trust, enabling the sharing of patient data by consent and (to the extent that any consent is dynamic), refreshing or updating consent when required. The way in which primary care provision is organized means that a large number of GP data controllers would be involved, whose views on whether and how to respond to a GP data trust are likely to differ.

#### 3. Identifying appropriate technical systems to support access to the data

We carried out a detailed exploration of the technical requirements for a GP data trust. This work included assessing possible means of accessing data from GP systems and either transferring them into the ultimate GPDPR data repository or ensuring that, wherever the data were held, patients’ sharing preferences (expressed by some form (ideally dynamic) of meta-consent given when contributing data) could be acted upon. We also considered the technical requirements for a GP data trust, technical possibilities for withdrawing consent for participation, and ensuring full transparency of data use to the beneficiaries of the data trust. The conclusions of the technical work are reported elsewhere.^32^ Our aim here is to explore a possible operational and governance model for a GP data trust.

#### 4. Identifying the appropriate operating model

A key aspect of the work undertaken in the GPDT study, using findings from the empirical research, was to establish the most appropriate data stewardship governance model for a GP data trust. Initial indications from other research^33^ suggested that a trust model would be appropriate, where a legal relationship is created to place assets under the control of a trustee for the benefit of a beneficiary or group of beneficiaries.^34^ Trusts combine the representation of beneficiaries’ interests and wishes with trustee oversight and accountability (including, crucially, the creation of fiduciary duties and responsibilities). Finally, and by no means least important, was how a GP data trust might be funded. Possible options included member subscription or charging for data access.

These questions were explored in more depth through surveys, interviews, and focus groups.

### B. Methods and findings of the GPDT study

A variety of methods were used to explore the challenges identified above. We conducted surveys of patients (*n* = 184 responses) and general practitioners (*n* = 86 responses). The survey data were further explored in qualitative semi-structured interviews with patients (*n* = 10), general practitioners and practice managers (*n* = 2), and medical researchers and representatives of other interested groups, including campaign groups, patient representatives, and others working to find solutions to the problem of health data sharing (*n* = 6). Furthermore, two online focus groups were held with *n* = 22 participants in total.

Understanding the views of patients who had decided to opt out of the GPDPR programme was crucial for ensuring greater patient participation in any alternative data-sharing model. Equally crucial was a better understanding of the reasons underlying GPs’ concerns. Noting that some GPs had recommended that all of their patients opt out, we felt that interviewing GPs to explore whether a GP data trust might overcome their concerns would also be helpful. While a number of GPs expressed interest in being interviewed, due to their workload pressures, it was not possible to speak with them during the time available for the study. Our data were therefore predominantly gathered from qualitative engagement with patients or representatives of other interested groups, such as health researchers and policymakers.

#### 1. Surveys and interviews

Patients were recruited through a social media campaign, local press, professional contacts, and existing relationships. GPs were recruited by Qualtrics’ Online Panel. GP and patient participants were asked to complete an online survey.^35^ After completing the survey, participants were asked to indicate their willingness to take part in a follow-up interview (it was also open to GP and patient participants to choose to be interviewed without completing a survey). A proportion of those who indicated their willingness to take part in an interview were contacted directly by email. Consent to participate was obtained, and all interviews were conducted and recorded using secure video conferencing (Zoom). The audio files were sent to a transcription service, and transcripts were checked for accuracy against the audio recordings before being anonymized.

We conducted *n* = 18 interviews with people from a range of stakeholder organizations (*n* = 6), including campaign groups, patient organizations (*n* = 10 patients interviewed), and other people working in data stewardship. The interviews were conducted according to a semi-structured interview schedule and lasted for around an hour. The focus of the interviews was on identifying and exploring the participants’ views on the sharing of GP data as anticipated by the GPDPR programme. The interviewer (C.A.B.R.) aimed to elicit participants’ concerns, and how, from the participant’s perspective, those concerns might be addressed. Interview participants were recruited until no new themes were identified from two consecutive interviews.

#### 2. Focus groups

Two online focus groups were held. Participants for the focus groups were found through an appeal in the Patients Association’s weekly email newsletter. As a result, participants skewed towards people with above-average levels of insight and knowledge on healthcare, either from professional experience or active personal interest. At least 10 of the participants identified themselves as, variously, qualified or trainee health or social care professionals, professionals in related fields, members of health or care-related organizations, or as occupying various other professional or voluntary roles in the health and care system. We therefore acknowledge the possibility that some views that emerged in the focus groups may have been held more strongly, or more commonly, than in the general patient population.

A total of 53 people expressed interest in participating, of whom 40 were telephoned. Twenty-four were selected to give a diverse representation of age, gender (a 50–50 split, including one transgender woman), ethnicity, and UK geographic region. There was a mixture of people who had opted out of sharing their health data and those who had not. Some were unsure whether they had opted out, and others had tried to but it became apparent that they had not. Those who were unsure about whether they had opted out were asked what they thought they would have chosen to do at the time, or what they would choose to do now, so that participants could be classified as those who opted out or would have opted out, or those who did not or would not have done. An approximately 50–50 split was then allocated to the focus groups.^36^

The focus group discussions coalesced around descriptions of a pervading climate of mistrust, underpinned by participants' personal experiences and of others known to them, and exacerbated by the failures of the care.data initiative and the GPDPR programme, which was described by one participant as having been done in, ‘*I think the word’s [a] suspicious manner, it was like a thief coming into the house or a fox into the hen coop, with no publicity.*’^37^ Reasons for the mistrust were varied but, for some, were rooted in concerns about fundamental changes to the NHS. ‘*There is a fear of creeping privatisation within the National Health Service and there is evidence that it is going on*.’^38^ A related concern was the growing role of private companies in providing NHS services and the sharing of patient data with such organizations:

*What I'm concerned about is that…there was an article in The Guardian recently which talked about the government is about to award a contract to an American company, which will actually hoover up our data, regardless of whether we give consent or not, and will be able to use it…*^39^

A significant concern of focus group participants was the profit motives of such private companies, which were invariably referenced in implicitly or explicitly negative terms, with profit-driven motivation always being perceived negatively. Contrasts were often drawn between the NHS’s focus on public benefit (treating everyone, undertaking research) and the harmful behaviours by private organizations (making profits, behaving unethically). These perceptions were explicitly linked to reservations about sharing personal data:

*I’m very happy to share my data with NHS trusts around the country, so that wherever I might fall ill, they have access to my records. Beyond that, I certainly wouldn’t want private companies having any access.*^40^

*I don’t think I would be happy for private companies, for example, to have access, or anyone who’s going to make a profit of my data.*^41^

Further, the importance of trust and transparency was clear, with participants identifying the lack of these key features as the primary reasons for which the GPDPR programme failed. Also important was the assurance that patients would have a level of control over how and with whom their data were shared, and the need for mechanisms of oversight and redress to be built into the organizational model. These things, together with the importance of a clearly (and transparently) quantifiable value or defined benefit being linked to the purposes for which GP data would be shared, described a series of ‘red lines’ by reference to which we have considered the key features of the organizational model we propose. Participants’ specific feelings about the idea of a data trust were also relevant to these deliberations, including the desirability of a data trust that was controlled by patients (‘*the board should be comprised mostly of the people that the data is going to be drawn from as much as possible*’^42^), although the complexity of striking a balance between this and having appropriate clinical and technical input into trust governance was acknowledged. While generally supportive of the data trust concept, participants did express concerns, including that a data trust might become ‘*another little entity that is doing its own thing’*, become just another ‘*little silo*’ or be managed by ‘*a compliant board of trustees*’.^43^

In general, participants were positive about sharing their data. The many benefits, in the healthcare context, of the availability of patient data were mentioned, but, as a counterpoint, certain pre-requisites to sharing were identified, including the need for transparency to underpin informed consent and choice. There was strong support for the use of patient data in health research, with a preference for research that benefited the NHS and an aversion to research involving private companies. It was for these reasons, underpinned by participants’ altruistic reasons for sharing health data, that we decided to consider the use of a charitable vehicle for a GP data trust.

## IV. A CHARITABLE DATA TRUST FOR GP HEALTH DATA?

The findings of the GPDT study indicate that notions of altruism and of ‘giving back’ to the NHS were important to participants in deciding whether or not to consent to their health data being shared:

*So it should be not-for-profit or can they make a profit that goes back into the NHS, so if…because if it’s not-for-profit and these pharmacy companies are making loads on the back of it, then I would want [the NHS] to have their share* [130 Interview]

*If I sell my data to […] another third-party company that is spamming me with marketing, I’m not happy. But for the benefit of the population health, I’m very happy that they use my data, that’s fine.* [177 interview]

*I wouldn’t mind to share my information if it helped some other…helps the NHS or it helps to develop, for example, the study you’re doing today. If that can benefit… If my experience, my record helps other fellow human beings, then, no, I wouldn’t have anything to object to.* [195 interview]

*I’ll be generous because I have been benefitting from the NHS, not because I work for it but because I’m a service user as well so in order to… If there is a way the NHS can save money or maybe earn money, yes, I’ll be more than happy to share this information because, at the end of the day, we all need this care. We don’t know what lies five years down the line but I want the NHS to stay so, therefore, people can be benefitted. So, yes, if my little tick-box consent can help or bring some benefits, why not?* [195 interview]

The views expressed by the participants indicated that the operational model governing the sharing of their health data should be both trustworthy and afford individual patients some degree of control in such sharing. Participants strongly favoured a model that would facilitate the flow of benefits to the entire body of NHS stakeholders (comprising patients, the public, and NHS healthcare professionals), and, specifically, not just to the shareholders of ‘big pharma’ companies. Furthermore, and of central importance to the data trusts model, is that ‘data trustees’ be bound by fiduciary duties to act in good faith in the interests of the trust’s beneficiaries. Before proceeding to consider the potential benefits of a charitable vehicle, we briefly describe the key features of a corporate vehicle and indicate why a non-charitable corporate vehicle would have unacceptable limitations in light of these requirements.

###  

#### 1. The limited company—a brief introduction

A company is a ‘body corporate’ comprising the members of the company from time to time.^44^ A company has its own existence and ‘legal personality’, which means the law considers it a person with the ability to exercise various rights and powers, as well as to be bound by obligations and liabilities.^45^ This means a company can enter into contracts, own land and property, employ staff, take legal action against other people, and be sued itself. A company acts through its directors, who are its agents. Where a company has shareholders, they provide funds to the company through the payments they make for their shares. Shareholders are entitled to their share of any profits the company makes, but they do not own (and are not entitled to) any of the company’s property. The company itself is the legal owner of its land and property; assets are not held on trust for its shareholders.

The position of company directors, vis-a-vis the company, is akin to that of trustees, in that they owe trustee-like duties to the company. Being entrusted with the management of the company’s affairs, directors are required to give it their undivided loyalty.^46^ In addition, the Companies Act 2006 codified some of the common law and equitable duties of company directors.^47^ Briefly, these duties obligate company directors: to act within their powers^48^; to promote the success of the company^49^; to exercise independent judgment^50^; care, skill, and diligence^51^; to avoid conflicts of interest^52^; to declare personal interests in certain aspects of the company’s business^53^; and not to accept benefits from third parties.^54^ These duties are owed *to the company*, though, and not to its members.^55^

For the majority of companies with shareholders, success is generally closely linked to a company’s financial success and the generation of market share, profit, or growth. Companies without shareholders may measure success in different ways, such as, for instance, by reference to the achievement of community or social objectives. In these cases, profits may (but do not always have to^56^) be reinvested to support the achievement of those objectives. However, for all companies other than those registered as charities, the company itself is the beneficiary of its directors’ fiduciary duties, even where it has social or community objectives.

Thus, as a vehicle for a health data trust, a non-charitable limited company is unlikely to meet the requirements of participants in the GPDT study. Jessica Bell’s recent analysis supports our view that charity law offers an appropriate framework for the management of data for public benefit purposes.^57^ Our use of empirical data to underpin a discussion of the governance mechanisms set out in the model constitution for a CIO (rather than a limited company) builds on this work. We now turn to a more detailed consideration of charitable models within the context of GP data sharing.

#### 2. The legal requirements for recognition as a charity

A charitable data trust would have to comply with the general legal requirements for charitable status. Below, we describe these requirements, consider how a GP data trust might meet them, and then, assuming that it does so, go on to consider which type of charitable vehicle might be appropriate. Working through the key features identified by our participants, we next outline how the constitution of our proposed vehicle could support them in practice.

#### 3. Charitable purpose

To be recognized as a charity in law, an organization must be established for exclusively charitable purposes.^58^ A charitable purpose is one that both falls within a list of 13 potentially charitable purposes set out in the Charities Act 2011 and is exclusively for the public benefit.^59^ The most relevant of these 13 potential purposes in this context is the advancement of health or the saving of lives, where ‘the advancement of health’ includes the prevention or relief of sickness, disease, or human suffering.^60^

Charity Commission guidance describes the ‘advancement of health’ broadly:

The advancement of health includes the prevention or relief of sickness, disease or human suffering, as well as the promotion of health. It includes conventional methods as well as complementary, alternative or holistic methods which are concerned with healing mind, body and spirit in the alleviation of symptoms and the cure of illness. […] The relief of sickness extends beyond the treatment or provision of care, such as a hospital, to the provision of items, services and facilities to ease the suffering or assist the recovery of people who are sick, convalescent, disabled or infirm or to provide comforts for patients.^61^

Medical research charities, as well as charities promoting activities that have a proven beneficial effect on health, are specifically listed in the guidance as examples of the sorts of charities and charitable purposes falling within this description. Looking at the constitutional documents of active charities in this field, it seems uncontroversial to suggest that (assuming its activities are for the public benefit) a GP data trust whose purposes were described as (for example):

To promote and support the use of NHS general practice health data for medical research and, in particular, for research, training, public engagement and dissemination of knowledge with the aim of improving human health by improving diagnosis, healthcare, treatments and planning of NHS services on terms acceptable to patients would be accepted for registration by the Charity Commission.^62^

#### 4. Public benefit

In addition to falling within the ‘advancement of health’ purpose, the activities of the GP data trust would have to be carried out for the public benefit if they are to be charitable.^63^ Much court time has been spent, and ink spilled, considering the legal meaning of the public benefit requirement, which has been an essential element of the legal concept of ‘charity’ since the first description of charitable purposes in the preamble to the 1601 Statute of Elizabeth. The courts have not, however, attempted to reduce the body of common law to a comprehensive statement of the meaning of ‘public benefit’, and neither is there a statutory definition of the term.^64^ The complexities of the case law on public benefit, the risk of legal challenge and unintended consequences, and the inflexibility of a ‘one size fits all’ statement or statutory definition compared to case law, have (to date) all mitigated against both of these things. However, the Charity Commission has published detailed guidance regarding the scope of activities likely to be acceptable for each of the 2011 Act’s 13 potentially charitable purposes.^65^ This guidance underpins our analysis below of how the activities of a GP data trust might fulfil the public benefit requirement.

A GP data trust’s activities would relate to the establishment, maintenance, and promotion of a resource for research that could be as widely drawn as to protect, preserve, and advance all or any aspects of the health and welfare of human beings and, possibly, also to advance and promote knowledge and education.^66^ A subset of such a very widely drawn object could make specific reference to supporting the planning and delivery of NHS service provision, as well as the promotion of health throughout society for the benefit of NHS stakeholders (patients, staff, and the general public).^67^ The beneficiaries of a charitable institution are the people or organizations that fall within the class of people who will or may be helped by the charity.^68^ The potential beneficiaries of the GP data trust would, therefore, comprise the general public at large, including all stakeholders of the NHS. Thus, the pool of potential beneficiaries of a charitable GP data trust would not be limited to those patients whose GP data were included in the databank. Their charitable agency would deliver benefits to a wider public, potentially (as the objects of UK Biobank Limited aim to do) to humankind.^69^ This approach would meet both the requirements of the research participants and sit comfortably with the organization of the NHS around principles of solidarity and mutual benefit.^70^

Assuming, for the reasons briefly summarized above, that a GP data trust would be accepted for registration as a charity (and that a charitable model would be acceptable to the patients whose health data comprise the assets of the charity), we next consider what charitable structure would best support the proposed activities and ownership of the data trust.

#### 5. Choosing a charitable structure for a GP data trust

There are four common charity structures, divided into two main groups. These are incorporated charities, which have legal personality^71^ (consisting of charitable companies and CIOs), and unincorporated charities (either charitable trusts or charitable associations). An unincorporated charity has no separate legal personality and is not a legal entity in its own right. This means that the charity itself cannot hold property or other assets, nor can it enter into contracts or other obligations. Accordingly, due to the complexity of the activities, a GP data trust would carry out (including, eg, entering into the contractual relationships that would underpin the way access would be granted to the dataset) a limited liability corporate body, whether a charitable company or a CIO, would, in our view, be the more appropriate vehicle, both legally and practically.

Many incorporated charities are companies, regulated by Companies House and subject to the laws applying to all limited companies. These are typically limited by guarantee, rather than by shares. This means that members of the company have no shareholding (and therefore no property interest) in the company and, rather than being distributed to members, any profits are re-invested into the charity and used to support its activities. As well as being subject to company law and regulation by Companies House, charitable companies are also subject to charity law and regulated by the Charity Commission. While the dual regulation does add some complexity to the operation of charitable companies, the charitable company structure has been available for many years, and the company law framework in which limited companies operate is well-established and understood.^72^ This can be a particular advantage when negotiating significant contracts, as well as for commercial lending or investments. In contrast, the CIO, a corporate structure designed specifically and exclusively for charities, was introduced only in 2013.^73^ Like a charitable company, a CIO is a limited liability corporate body. It has legal personality and, thus, the ability to enter into contracts and hold property in its own name. Unlike a charitable company, however, a CIO is registered with and regulated solely by the Charity Commission, coming into existence only when its details are entered into the register of charities. CIOs also have the same two-tier trustee/member structure as charitable companies, and the liability of members is limited in the same way. This means that if the CIO is wound up, its members are either liable to contribute (up to) a specified amount to the assets of a CIO or required to make no financial contribution at all (depending on the basis on which they agreed to become a member).^74^ Furthermore, both members and trustees of CIOs have statutory duties to exercise their powers in a way that they consider, acting in good faith, is most likely to further the purposes of the CIO.^75^

The CIO was designed specifically and exclusively for use by charitable organizations. The anticipated benefits of the new structure were that CIOs would offer limited liability for charities while avoiding the burdens of dual registration, provide a governance structure that was not built around members’ financial interests, and clarify the overlap between the differing duties imposed by corporate and charity law on directors of charitable companies.^76^ Legal personality and limited liability are attractive to charities whose activities involve risk and complexity, such as using or owning land, employing staff, and entering into significant contractual liabilities and funding arrangements. Oversight by a single regulatory body, the Charity Commission, simplifies (and reduces the cost of) setup and administration for CIOs compared to charitable companies (with their dual registration and regulation requirements). However, there are limitations to the CIO model, such as the lack of a web-based, searchable register of charges over the property of a CIO.^77^ This means that, for charities that routinely seek to borrow money against the security of their property (such as land-owning charities), a CIO might be a less attractive option. In our view, this would not constitute a significant concern for a GP data trust.

Thus, subject to further exploration of the issues of the technical structures, sustainability, and funding matters, we consider that the use of a CIO for a GP data trust shows great promise in its potential to respond to patient concerns about the sharing of their health data. In the next section, we consider the CIO constitution and governance generally,^78^ focusing, as we do so, on the ‘red line’ requirements of participants in the GPDT study: that patients have a level of control over how, with whom, and for what purpose their data are shared; that trustees (understood as those responsible for running the data trust) have clear responsibilities to the patients whose data they manage (fiduciary duties being a good way of achieving this); that the benefits of sharing the data do not flow (solely) to ‘big pharma’ companies; and, finally, that mechanisms of oversight and redress are available in the event that things go wrong.

#### 6. The constitution and governance of a CIO

All CIOs are required to have at least one charity trustee and at least one member.^79^ They must also have a constitution, as close in form as possible to one of the two model constitutions published by the Charity Commission.^80^ These model constitutions describe differing governance structures: a ‘foundation’ model, where all members are charity trustees and vice versa, and an ‘association’ model, which in our view is more appropriate for a GP data trust, as it has a voting membership wider than its charity trustees. In both models, the trustees have ultimate legal responsibility for strategic leadership and remain responsible for the day-to-day management and administration of the charity.

In the sections that follow, we consider aspects of the constitution and governance of an association CIO, highlighting specific features that, from the perspective of our participants, appear to suggest that this charitable vehicle has potential for use as a GP data trust.

#### 7. Association CIO members, their powers, and responsibilities

Any individual or corporate person may be a member of an association CIO, and we are not anticipating that GP data trust members would necessarily be restricted to those patients who have contributed their data to the data trust, or, conversely, that patients who contribute their data would have to be members. CIO membership is generally open to anyone interested in furthering the purposes of the CIO,^81^ so in a GP data trust, members might include patients, GPs, and, potentially, GP practices^82^ or other organizations. It would be open to a GP data trust to restrict membership, if it were considered reasonable to do so.^83^ This might mean, for example, excluding pharmaceutical companies from membership—although it would also be open to the GP data trust to establish different classes of voting membership^84^ and to charge membership fees,^85^ which might be a more productive way to ensure the engagement of the pharmaceutical industry in the GP data trust. Membership can be terminated in various ways, including where the trustees decide that the best interests of the CIO would be served by removing a particular member, in which case a fair process is mandated.^86^ This process is managed by the trustees but, were the members to oppose the proposed removal, it would be open to them to request a proposal for a decision on the matter by the members.^87^

The voting membership of an association CIO has rights and powers to participate in the governance of the charity, including the right to appoint some or all of the charity trustees, the right to amend the CIO’s constitution (subject, in some cases, to Charity Commission approval), and the right to close the CIO down.^88^ In addition, the voting members provide a layer of internal scrutiny and accountability in relation to the activities and management of the CIO. However, the members of a CIO may not exercise the powers of the CIO; that is to say, they would not have responsibility for its day-to-day management and administration, such as, for instance, managing the data trust’s employees or negotiating the contracts pursuant to which the GP data would be shared, save to the extent that they were appointed to specific committees of the CIO with decision-making powers (as to which, see further below).^89^ The day-to-day management of the data trust would be the responsibility of an executive team, managed by and accountable to the trustees. However, the model constitution for association CIOs gives members the power to appoint trustees and to remove them (in certain circumstances, and subject to a fair process).^90^

Legal members of a GP data trust would therefore have a limited but important role as a group of decision-makers. They would be required to exercise independent judgment in deciding how to cast their vote and to vote in the way that they, in good faith, consider would best further the interests of the CIO.^91^ The members’ statutory duty is a subjective duty—such that, for example, in voting for one trustee candidate over another, each member must genuinely believe the appointment of their preferred candidate would best further the GP data trust’s charitable aims.^92^ To an extent, the CIO’s charitable purpose, and the members’ statutory duties in respect of trustee appointment, act as checks on the ability of a vocal minority of trustees to dominate the board, a concern expressed by the research participants (as to which, see further below). Were this to happen, however, procedures exist, also underpinned by a focus on the CIO’s best interests, which allow for their removal.

The members described above are legal, voting members of the CIO, bearing the rights and obligations imposed by charity law and regulation. It is also possible for an association CIO to create informal or associate, non-voting classes of membership, without any of these legal rights and obligations.^93^ For patients (or others) who do not want to engage in the GP data trust as legal members, participation as associate members could be an option. It would be open to the GP data trust to determine the rights and obligations of such informal members—options might, for instance, include a patient-engagement-style membership (which might be supported by, or affiliated with, eg, Healthwatch or the Patients Association).^94^ Alternatively, a GP data trust might choose to make strategic use of classes of informal or associate membership so that certain organizations (such as charities supporting research into particular diseases) could become affiliated with the data trust. This might facilitate frictionless access to appropriate data for research, but also serve as the nexus of support for patients suffering from a particular condition, or as a route to identifying potential participants in clinical trials, or even co-producing research of priority interest to data trust members. In becoming associate members, organizations might be asked to comply with certain conditions, such as, for example, affordable licensing of any resulting intellectual property to the NHS. It would be open for associate members to sit on Committees of the GP data trust (with trustees and legal members) to include relevant voices in GP data trust decision-making.

An association CIO GP data trust could, in these ways, enable (but not require) patient and other interested parties’ engagement to greater or lesser degrees as members of the charity. We now move to consider the roles and responsibilities of the trustees of an association model CIO, again with reference, where relevant, to the GP data trust.

#### 8. The roles and responsibilities of charity trustees in a GP data trust

Charity trustees have significant legal duties and, if they fail to discharge those duties, potential legal liabilities.^95^ The charity trustees’ function is to manage the affairs of the CIO, exercising the powers of the CIO^96^ to further its objects.^97^ In exercising those powers, trustees must act in good faith and with reasonable skill and care.^98^ What is ‘reasonable’ for these purposes depends on the special knowledge or experience each trustee has—so that trustees with particular professional or business expertise would be required to apply any special knowledge or experience that it would be reasonable to expect them to have. This duty mirrors the duty of care for trustees generally (set out in the Trustee Act 2000).^99^ There are various checks and balances built into the model constitution to guard against a minority of trustees dominating the running of the CIO. These include the ability to specify particular classes of trustee, to set limited terms of office, and require retirement by rotation, as well as allowing for both appointment of trustees (by the board) and election by the membership.^100^ The constitution also sets out a process for the removal of trustees.^101^ Furthermore, the use of committees of the board (described further below) offers a mechanism to distribute decision-making more widely and reduce the possibility that the trustees, or a vocal minority, could take the CIO in a direction unpopular with its members.^102^

Key to the aims of a data trust, as we have seen, is the fiduciary nature of trusteeship, which applies to all charity trustees, irrespective of the legal nature of the charity for which they are responsible. Fiduciary duties coalesce around a core obligation of loyalty, which a charity trustee owes to the charity’s beneficiaries. This duty obliges trustees to ensure they do not, unless specifically authorized to do so, put themselves in a position where their personal interests conflict with their fiduciary duties, or where there is a real possibility of such conflict. For this reason, charity trustees are prohibited from making any profit from their fiduciary office, and charity trustees are generally volunteers, who are not paid for taking on this role. The prohibition on making a profit can be relaxed in some clearly defined circumstances. For instance, the model constitution permits a minority of the trustees to receive payments for any goods and services they supply to the CIO, provided these payments are properly authorized.^103^ It is also permissible for trustees to be employees of the CIO, providing that employing the trustee is in the interests of the charity, and that the constitution both expressly permits trustees to be employees and contains a process for managing the resulting conflict of interest.^104^ Trustees may also benefit from the activities of the charity as *beneficiaries*, provided that this benefit is available generally to all beneficiaries of the CIO. In the context of a GP data trust, trustees would, as stakeholders in the NHS (whether as staff, patients, or the general public),^105^ also be beneficiaries of its charitable activities, and, thus, the CIO’s constitution would need (as the association model constitution does) to expressly permit such a benefit.^106^

The model constitution for association CIOs contains detailed provisions (including various options and alternatives) concerning the eligibility of trustees, their number, their appointment, retirement, and removal, as well as the way trustee decisions should be made.^107^ Members of the CIO may be appointed as trustees. The model Constitution also permits trustees to delegate their powers (including to make decisions) to a committee or committees.^108^ Importantly, this power of delegation goes beyond that afforded to trustees generally under the Trustee Act 2000. Under that Act, trustees may only delegate a power to *carry out* a decision they have already taken, rather than to *make* decisions on behalf of trustees.^109^ Appropriately constituted committees, with clear terms of reference and delegated powers, could offer a sensible starting point for detailed and specific oversight over aspects of the data trust’s operations, for example, access to the GP data trust’s dataset(s). Members (legal and informal alike) would be able to serve on committees alongside trustees and others whose expertise might be useful. Committees must have at least two members, of whom at least one must be a charity trustee, and are subject to trustee review.^110^ In forming committees, trustees would have to give careful thought to how decisions are made, if this power is to be delegated, including quorum, casting votes, and how to resolve any differences of opinion. It might be helpful to ensure that all members are reminded that decisions reached at a committee meeting are collective decisions and, thus, binding upon all committee members.^111^

Appropriately constituted committees might also make decisions on researcher approval, arrangements for accessing data, and access fees. Fees could be set to reflect the concerns of members around commercial usage of the data, so that commercial players might pay a higher access fee, to the benefit of the data trust going forward. Transparency might be provided by the formulation of a fee structure. Charitable status would not affect the data trust’s ability to charge for access to the data, provided that the activities of the data trust were for the public benefit.^112^ This might require that researchers be obliged to publish their findings on an open access basis, and/or provide the NHS (and/or the data Trust) with access to their results. Licenses for NHS use of (or access to) intellectual property might also be part of the arrangement (although there are plenty of questions related to intellectual property licensing that would need further exploration). The resulting funding stream would enable the data trust to invest in maintaining and improving the primary care databank, including ensuring that technical and privacy functions remained appropriate and state-of-the-art, such that patients’ trust in, and continued engagement with, the data trust could be maintained.

Finally, addressing the concern of participants in the GPDT study that trustees be accountable to the GP data trust, trustees of a CIO, like directors of a charitable company, may be personally liable under criminal and civil law in certain limited circumstances. Trustees may be held to account if they receive unauthorized payments or other benefits from the CIO, in which case they may be required to repay the value of any such benefits to the CIO. In addition, they may be held liable for any actions in breach of their duties where loss has been caused to the CIO as a result. In practice, where a trustee has acted honestly, reasonably, and in good faith, it is unlikely that they will be held personally liable for such breaches.^113^ In an association CIO, an added level of reassurance lies in one of the roles of its voting members being to oversee and scrutinize the actions of the trustees.

For the reasons discussed above, we consider that a (or the) GP data trust could be constituted as a charity. Its core activities (supporting its charitable purpose) would be to make the GP dataset available (only) to bona fide, approved researchers for health-related research in the public interest. The association CIO model, within the general charity law framework and the more specific context of regulations applying to CIOs, meets the requirements identified by participants in the GPDT study, particularly as regards fiduciary responsibility, the ability for patients to exercise a reasonable measure of control over the sharing of their data, and to have oversight over (and some involvement in) the actions and decisions of trustees. In the final section, we sketch out some ‘bigger picture’ thoughts and call for further pilot work to test our suggestions in a practical context.

## V. A CHARITABLE GP DATA TRUST AND THE BIGGER PICTURE

Our purpose in this article has been to draw on the findings of the GPDT study in considering how a GP data trust might be constituted to reflect the preferences of patients in relation to their health data sharing, and to highlight areas for further inquiry rather than suggesting a definitive way forward. Our view is that a charitable GP data trust is a model that warrants further empirical and practical exploration. In concluding, we offer some wider reflections, noting, in particular, the congruence of a charitable vehicle and the communitarian underpinnings of the NHS.^114^

The CIO was clearly not designed for use as a national corporate custodian of GP data. However, as we have suggested in the discussion above, there is no unsurmountable conceptual difficulty in the use of a CIO for a data trust of health data. Where, in practice, we do see an issue is that CIOs are intended to be directly regulated by the Charity Commission, which is likely to lack the resources to oversee an organization with the size and potential complexity of a GP data trust. Our first additional reflection, to address this problem, is to suggest the possibility that a charitable GP data trust might be an ‘exempt charity’, that is to say, exempted from registration with the Charity Commission and overseen by another appropriately qualified regulator. Exempt charities have charitable status and must comply with general charity law but are not registered with, or directly regulated by, the Charity Commission.^115^ Most English Universities are exempt charities, for example, as are a number of museums and other institutions of national importance.^116^ These universities are regulated by the Office for Students, and museums and galleries by the Department for Culture, Media, and Sport. Suggesting that a GP data trust be an exempt charity immediately then raises a question about the identity of the organization best placed (and resourced) to regulate its compliance with charity law. While we do not presume to answer this question here, we note that NHS England continues to review primary care data sharing and has recently amalgamated with the former NHS Digital. However, were NHS England to take on such a role, there would no doubt be concerns raised about regulation of the ‘new’ model by an organization in whom public trust and confidence have been lost as part of the GPDPR fiasco. In addition to identifying the principal regulator, specific and appropriate regulation would clearly be required for any GP data trust to be exempted from registration with the Charity Commission,^117^ but a regulatory project of the size and scope of the GPDPR project could push for such a move.

Indeed, it is arguable that a regulatory project of the size and scope of the GPDPR project is comparable in scale to the creation of the NHS in the first place. Shifting power over their data from ‘the system’ to individual patients involves a conceptual and philosophical shift in thinking similar to that which Aneurin Bevan, generally recognized as the founder of the NHS, championed when he wrote about the creation of a national health service in the 1950s.^118^ He sought, in creating the NHS, to displace the predominantly market-based model of healthcare that then prevailed with one based on communitarian values, arguing that ‘medical treatment and care should be a communal responsibility; that they should be made available to rich and poor alike in accordance with medical need and by no other criteria’.^119^ The current NHS Constitution reflects these ideas and was intended to embed the principle of stakeholder involvement into the NHS, and evoke a sense of shared ownership of it.^120^ For patients, for example, the NHS Constitution was supposed to provoke challenge and shared responsibility for making the best use of NHS services.^121^ The public as a whole has a stake in the NHS, and particularly in the aftermath of the coronavirus disease 2019 (COVID-19) pandemic (as one of us has argued elsewhere^122^), operationalization of our responsibilities as NHS stakeholders encompasses participating in a conversation, on a society-wide level, about how we should understand and engage our rights and responsibilities under the NHS Constitution.^123^

In a sense, by requiring involvement in decisions about the use of their GP health data, the GPDT study participants reflect this sense of engagement with their rights as NHS stakeholders. In suggesting that they want health data research to give back to the NHS, they are exercising the responsibilities expected of them as NHS stakeholders. Ben Goldacre, reflecting on the value of 73 years of NHS patient records, containing ‘all the noise from millions of lifetimes’ and representing ‘deeply buried treasure that can help prevent suffering and death around the planet on a biblical scale’, concludes that it is our collective duty to make this work.^124^ Furthermore, the advent of big data and AI means that radical re-evaluation of patients’ roles in the sharing of their data is required (including a rethink about whose it is). Operationalizing the concept of the GP data trust offers an opportunity to do just that, involving patients in decisions about the use of their data in health research and ensuring that their interests are at the heart of what is done with it. We suggest, then, that the notion of a charitable GP data trust offers all of us, whether or not we have spoken up to date about the use of our GP health data, the opportunity to engage in this exciting and timely project.

## Ethics

Ethics approval for the General Practice Data Trust pilot study was received from The University of Manchester Ethics Committee, ref 2022-15218-25912.

## Footnotes

1 Ben Goldacre and Jessica Morley, *Better, Broader, Safer: Using Health Data for Research and Analysis. (Goldacre Review): A Review Commissioned by the Secretary of State for Health and Social Care* (Department of Health and Social Care, 2022) 210.

2 ibid.

3 ibid.

4 Health Education England, NHS Digital and NHS England have merged into a single organization. For the former NHS Digital service, this means that NHS England has assumed responsibility for all activities previously undertaken by NHS Digital. This includes running the vital national IT systems that support health and social care, as well as the collection, analysis, publication, and dissemination of data generated by health and social care services, to improve outcomes.

5 Data Provision Notices (DPN) may be issued by NHS Digital pursuant to the statutory power to require and request the provision of information under ss 259(1)(a) and 259(1)(b) of the Health and Social Care Act 2012.

6 NHS Digital, *Data Provision Notice: General Practice Data for Planning and Research* (Health and Social Care Information Centre 2021).

7 ibid 4.

8
*The Guardian* <www.theguardian.com/society/2021/aug/22/nhs-data-grab-on-hold-as-millions-opt-out>; *Byline Times* <https://bylinetimes.com/2021/05/19/the-government-wants-to-sell-your-gp-medical-records-heres-how-to-opt-out/> and <https://bylinetimes.com/2021/06/02/data-and-deception-the-governments-grab-of-gp-records-violates-our-trust/> all accessed 16 February 2024.

9 The term data subject has unfortunate, but perhaps appropriate, connotations of royalty and feudal society, where an individual is subject to whims beyond their control: there is a power-asymmetry between the subjects and the controllers. Sylvie Delacroix and Neil Lawrence, ‘Bottom-up Data Trusts: Disturbing the ‘One Size Fits All’ Approach to Data Governance’ (2019) 9 Int Data Privacy Law <https://doi.org/10.1093/idpl/ipz014> accessed 16 February 2024.

10 NHS England, *About the GPDPR Programme* <https://digital.nhs.uk/data-and-information/data-collections-and-data-sets/data-collections/general-practice-data-for-planning-and-research/about-the-gpdpr-programme> (last updated 6 June 2024) accessed 26 November 2024.

11 ibid. At the time of writing, however, little progress seems to have been made.

12 Linda A Jones and others, ‘Public Opinion on Sharing Data From Health Services for Clinical and Research Purposes Without Explicit Consent: An Anonymous Online Survey in the UK’ (2022) BMJ Open <https://doi.org/10.1136/bmjopen-2021-057579>; Ipsos MORI Social Research Institute, *The One Way Mirror: Public Attitudes to Commercial Access to Health Data* (2016); Healthwatch, 'Patient data is crucial for the planning of health services—and so is building trust in how the NHS uses it’ (*Healthwatch*, 20 July 2021) <www.healthwatch.co.uk/news/2021-07-20/patient-data-crucial-planning-health-services-and-so-building-trust-how-nhs-uses-it> all accessed 26 November 2024.

13 The GP data trust pilot study is funded by the Data Trusts Initiative and led from The University of Manchester by Professor Sarah Devaney. The study received ethical approval from The University of Manchester Research Ethics Committee through its Proportionate Review process. Approval was granted on 16 September 22 for the interviews and surveys (Ref: 2022-15218-25272) and amended (10 November 22) to include the focus groups (UREC Amendment Ref: 2022-15218-25912). The project website is available at: <www.socialsciences.manchester.ac.uk/csep/research/projects/gpdt-pilot-study/> (accessed 26 September 2023).

14 The Open Data Institute, *Defining a Data Trust* (19 October 2018) <https://theodi.org/article/defining-a-data-trust/> accessed 16 February 2024.

15 For a full discussion, see Delacroix and Lawrence (n 9); Bart Van der Sloot and Esther Keymolen, ‘Can we Trust Trust-based Data Governance Models?’ (2022) 4 Data Policy e45. <https://doi.org/10.1017/dap.2022.36>

16 Wendy Hall and Jérôme Pesenti, *Growing the Artificial Intelligence Industry in the UK* (Department for Digital, Culture, Media & Sport and Department for Business, Energy & Industrial Strategy 2017).

17 See *Bristol & West Building Society v Mothew (t/a Stapley & Co)* [1988] Ch 1.

18 ibid, and see Open Data Institute, *What is a Data Trust?* <https://theodi.org/article/what-is-a-data-trust/> accessed 16 February 2024.

19 See S Devaney and others, with the patients association, *General Practice Data Trust (GPDT) Pilot Study: Report on Patient Focus Groups* <www.patients-association.org.uk/blog/gpdt-pilot-study-report> accessed 19 November 2024.

20 The GPDT pilot study was led by Dr Sarah Devaney, with Professor John Ainsworth, Dr Catherine Bowden, Dr Nigel Burns, Dr James Cunningham, Professor Søren Holm, and Dr Caroline Redhead.

21 NHS England, *NHS England Sets out the Next Steps of Public Awareness About Care.data* (October 2013) <www.england.nhs.uk/2013/10/care-data/>; Fiona Godlee, *What Can We Salvage from Care.data?* (2016) <doi.org/10.1136/bmj.i3907> accessed 16 February 2024.

22 And we note the concerns current at the time of writing about the award of NHS contracts to Palantir Technologies, an American software company. See, eg, <https://nopalantir.org.uk/> accessed 19 December 23.

23 Professor Goldacre is the Bennett professor of evidence-based medicine and director of the Bennett Institute for Applied Data Science at the University of Oxford. He was commissioned by the Government to undertake a review of questions of data sharing—the Goldacre Report (n 1).

24 Science and Technology Committee, *Oral Evidence: The Right to Privacy: Digital Data* (HC 97) 11 May 2022 Q232.

25 ibid.

26 The Association of the British Pharmaceutical Industry, *Unlocking the Promise of UK Health Data* (ABPI 2020).

27 ibid.

28 ibid 13.

29 The Association of the British Pharmaceutical Industry (n 26).

30 ibid.

31 See (n 4).

32 The University of Manchester, *General Practice Data Trust (GPDT) Pilot Study* <www.socialsciences.manchester.ac.uk/csep/research/projects/gpdt-pilot-study/> accessed 19 November 2024.

33 See, eg, the Civic Data Identity Platform <cdip.lancs.ac.uk/> accessed 16 February 2024.

34 See HMRC, *Introduction to Trusts: Legal Background—Trust Law: What is a Trust?* (TSEM1004 February 2024) <www.gov.uk/hmrc-internal-manuals/trusts-settlements-and-estates-manual/tsem1004> accessed 16 February 2024.

35 The survey data are not reported in this article, which focuses on the interview and focus group data. This means that the perspectives of GPs, not many of whom were available (due to pressures of work) to be interviewed, are under-represented, which largely reports the patient perspective.

36 See Devaney and others (n 19).

37 ibid 19.

38 ibid 21.

39 ibid 23.

40 ibid 24.

41 ibid.

42 ibid 51.

43 ibid 48 and 49, respectively.

44 Companies Act 2006, s 16(2).

45 See the definition of ‘person’ in Interpretation Act 1978 Schedule 1; See description of ‘legal persons’ in HMRC Internal VAT Registration manual <www.gov.uk/hmrc-internal-manuals/vat-registration-manual/vatreg02100> accessed 16 February 2024.

46
*Boulting v Association of Cinematograph Television and Allied Technicians* [1963] 2 Q.B. 606.

47 Companies Act 2006 ss 171–177.

48 ibid s 171.

49 ibid s 172.

50 ibid s 173.

51 ibid s 174.

52 ibid s 175.

53 ibid s 177.

54 ibid s 176.

55
*Sharp v Blank* [2019] EWHC 3096 (Ch).

56 See Companies Act 2006 s 60.

57 Jessica L Bell, ‘Governing Commercial Access to Health Data for Public Benefit: Charity Law Solutions’ (2020) 28 MLR 247 <doi.org/10.1093/medlaw/fwz022> accessed 16 February 2024.

58 Charities Act 2011 s 1.

59 ibid s 2.

60 ibid s 3(1)(d) and s 3(2)(b), respectively.

61 Charity Commission for England and Wales, *Guidance on Public Benefit* (2013) <www.gov.uk/government/publications/charitable-purposes/charitable-purposes#the-advancement-of-health-or-the-saving-of-lives> accessed 16 February 2024.

62 This suggested objects clause was based on the Articles of Association of Medical Research Foundation, a charitable company limited by guarantee, with company number 7366816 and charity number 1138223.

63 Charities Act 2011 s 4.

64 Cabinet Office, *Government Responses to: (1) The Public Administration Select Committee’s Third Report of 2013–14: The role of the Charity Commission and ‘public benefit’: Post-legislative scrutiny of the Charities Act 2006 (2) Lord Hodgson’s statutory review of the Charities Act 2006: Trusted and Independent, Giving charity back to charities.* (Cmd 8700 2013), See the discussion here: Government response to recommendations on the legal framework for charities—GOV.UK (www.gov.uk). For a fuller discussion, see Daniel Halliday and Michael Harding (eds), *Charity Law: Exploring the Concept of Public Benefit* (Routledge 2022).

65 Charity Commission, *Guidance, Charitable Purposes* (2013) <www.gov.uk/government/publications/charitable-purposes/charitable-purposes#the-advancement-of-health-or-the-saving-of-lives> accessed 16 February 2024.

66 See, eg, the charitable objects of UK Biobank Limited (Charity no 1101332) <https://register-of-charities.charitycommission.gov.uk/charity-search/-/charity-details/4004061/governing-document> accessed 16 February 2024.

67 The NHS Constitution describes its stakeholders as staff, patients, and the public. Department of Health and Social Care, *The NHS Constitution for England* (2015) <www.gov.uk/government/publications/the-nhs-constitution-for-england/the-nhs-constitution-for-england> accessed 16 February 2024.

68 See Fundraising Regulator Code of Practice, *Glossary* <www.fundraisingregulator.org.uk/code/glossary#glossary-code-b> accessed 16 February 2024.

69 See Charity Commission register of charities entry for UK Biobank Limited (Charity no 1101332) <https://register-of-charities.charitycommission.gov.uk/charity-search/-/charity-details/4004061/what-who-how-where> accessed 16 February 2024.

70 Redhead and others, ‘Relationships, Rights and Responsibilities: (Re)viewing the NHS Constitution for the Post-pandemic “New Normal”’ (2023) 31 MLR 83 <academic.oup.com/medlaw/article/31/1/83/6677202> accessed 26 November 2024.

71 See (n 45).

72 The UK Biobank is a charitable company limited by guarantee: <www.ukbiobank.ac.uk/learn-more-about-uk-biobank/governance> accessed 16 February 2024.

73 Charities Act 2011, pt 11, Charitable Incorporated Organisations.

74 ibid ss 205(2) and (3).

75 ibid ss 220, 221.

76 Cabinet Office Strategy Unit, *Private Action, Public Benefit: A Review of Charities and the Wider Non-profit Sector: Strategy Unit Report* (Strategy Unit 2002).

77 The only public register of charges over CIO property is, therefore, those charges that trigger registration with the Land Registry.

78 We note that the use of a charitable company would permit similar organization and operation. As noted above, charitable companies are subject to the operation of company law and regulation, which is not considered in this article.

79 Charities Act 2011 ss 206(2)(b) and 205(2)(c), respectively.

80 ibid s 206(5).

81 Charity Commission, *Constitution of a Charitable Incorporated Organisation with Voting Members Other Than its Charity Trustees (Association Model Constitution)’ (Constitution)* (2023) <https://assets.publishing.service.gov.uk/government/uploads/system/uploads/attachment_data/file/1076198/Association_Model_Constitution_280422.pdf> accessed 16 February 2024. para 9(1)(a).

82 We note that many GP practices are partnerships. While, technically, unincorporated organizations without legal personality are not able to be members of a CIO in their own right, the association model constitution (at clause 9(1)(a)) provides for an individual representing an unincorporated organization, such as a GP practice, to be a member.

83 Constitution, para 9 (including the marginal note).

84 Noting that, where the members of a CIO are to have different voting rights, the General Regulations require (at Regulation 13(9)) that the CIO’s constitution must state the voting rights that are to attach to each class of member.

85 Constitution, para 9(5).

86 Constitution, para 9(4).

87 Constitution, para 10(3)(d) and (e).

88 Constitution, paras 28 and 29.

89 Constitution, para 18.

90 Constitution, paras 13 and 15, respectively.

91 Charities Act 2011, s 220, reflected in the Constitution at para 9(3).

92
*Re Ethiopian Orthodox Tewahedo Church St Mary of Debre Tsion, London* [2020] EWHC 1493 (Ch). This case was the first time that a court has considered the duty of a CIO’s members under s 220 of Charities Act 2011.

93 See the Constitution, para 9(6).

94 The Patients Association is an independent patient charity (no 1006733) campaigning for improvements in health and social care for patients.

95 Charities Act 2011; Charity Commission, *The Essential Trustee: What You Need To Know, What You Need to Do* <https://assets.publishing.service.gov.uk/media/64b65c9b71749c001389ed26/CC3_feb20.pdf> accessed 16 February 2024.

96 Charities Act 2011 s 216(2).

97 Constitution, para 4.

98 Constitution, para 12.

99 Trustee Act 2000 s 1.

100 Constitution, paras 12 and 13.

101 ibid para 15.

102 While the aim would be to run a GP data trust in line with patients’ requirements, a patient would be able to withdraw their data should they choose to do so.

103 Constitution, para 6.

104 Charity Commission guidance OG515-9, *Trustees Becoming Employees* (2023) <https://ogs.charitycommission.gov.uk/OG515-4.docx> accessed 16 February 2024.

105 See NHS Constitution (n 67).

106 Constitution, para 5(2)(a)—the wording is in the alternative provision provided in the notes.

107 Constitution, paras 12ff.

108 ibid para 18.

109 Trustee Act 2000, s 11(3)(a), applied to CIOs by The Charitable Incorporated Organisations (General) Regulations 2012 (General Regulations), see Regulation 33. The additional power to delegate decision-making authority is set out in para 18(1) of the association model constitution.

110 Constitution, para 18.2.

111 See Charity Committee Guidance, *Charities and Meetings* (CC48) <www.gov.uk/government/publications/charities-and-meetings-cc48/charities-and-meetings> accessed 16 February 2024.

112 Note that charging for access to charitable services is problematic only in cases where the benefit is limited to those who pay for access, such that it is not public benefit for the purposes of Charities Act 2011. See, eg, *Independent Schools Council v Charity Commission* [2011] UKUT 421 (TCC), which found that a charitable, fee-paying school must ensure that the poor could benefit from their activities in a way that was more than minimal or tokenistic.

113 Charities Act 2011, s 191. There are other potential liabilities for criminal actions, fraud, or actions related to insolvency, but a detailed consideration of these is outside the scope of this article.

114 Aneurin Bevan, *In Place of Fear* (Heinemann 1952); Kenneth Veitch, ‘Obligation and the Changing Nature of Publicly Funded Healthcare’ (2018) 27 MLR 267; Redhead (n 70).

115 Charities Act 2011, s 30(2)(a).

116 See Charities Act 2011, Schedule 3, for a list.

117 Charities Act s 22.

118 Bevan, n 114

119 ibid 75.

120 Department of Health and Social Care, *Third Report on the Effect of the NHS Constitution* (Corporate Report 2019), Introduction, para 6.

121 ibid para 7.

122 Redhead and others (n 70).

123 Department of Health and Social Care (n 67).

124 Goldacre Review (n 1) 210.

